# Fluoroquinolone resistance among fecal extended spectrum βeta lactamases positive *Enterobacterales* isolates from children in Dar es Salaam, Tanzania

**DOI:** 10.1186/s12879-023-08086-2

**Published:** 2023-03-07

**Authors:** Upendo O. Kibwana, Joel Manyahi, Helene Heitmann Sandnes, Bjørn Blomberg, Stephen E. Mshana, Nina Langeland, Adam P. Roberts, Sabrina J. Moyo

**Affiliations:** 1 Department of Microbiology and Immunology, Muhimbili University of Health and Allied Sciences, Dar es Salaam, Tanzania; 2 Department of Clinical Science, University of Bergen, Bergen, Norway; 3 Norwegian National Advisory Unit On Tropical Infectious Diseases, Haukeland University Hospital, Bergen, Norway; 4 Department of Microbiology and Immunology, Catholic University of Health and Allied Sciences, Mwanza, Tanzania; 5 Department of Tropical Disease Biology, Liverpool School of Tropical Medicine, Liverpool, UK

**Keywords:** ESBL-PE, Fluoroquinolone resistance, Whole genome sequencing

## Abstract

**Background:**

Fluoroquinolones have been, and continue to be, routinely used for treatment of many bacterial infections. In recent years, most parts of the world have reported an increasing trend of fluoroquinolone resistant (FQR) Gram-negative bacteria.

**Methods:**

A cross-sectional study was conducted between March 2017 and July 2018 among children admitted due to fever to referral hospitals in Dar es Salaam, Tanzania. Rectal swabs were used to screen for carriage of extended-spectrum β-lactamase-producing *Enterobacterales* (ESBL-PE). ESBL-PE isolates were tested for quinolone resistance by disk diffusion method. Randomly selected fluroquinolone resistant isolates were characterized by using whole genome sequencing.

**Results:**

A total of 142 ESBL-PE archived isolates were tested for fluoroquinolone resistance. Overall phenotypic resistance to ciprofloxacin, levofloxacin and moxifloxacin was found in 68% (97/142). The highest resistance rate was seen among *Citrobacter* *spp*. (100%, 5/5), followed by *Klebsiella.* *pneumoniae* (76.1%; 35/46), *Escherichia* *coli* (65.6%; 42/64) and *Enterobacter* spp. (31.9%; 15/47). Whole genome sequencing (WGS) was performed on 42 fluoroquinolone resistant-ESBL producing isolates and revealed that 38/42; or 90.5%, of the isolates carried one or more plasmid mediated quinolone resistance (PMQR) genes. The most frequent PMQR genes were *aac(6’)-lb-cr* (74%; 31/42), followed by *qnrB1* (40%; 17/42), *oqx,* *qnrB6* and *qnS1.* Chromosomal mutations in g*yrA*, *parC* and *parE* were detected among 19/42 isolates, and all were in *E.* *coli.* Most of the *E*. *coli* isolates (17/20) had high MIC values of > 32 µg/ml for fluoroquinolones. In these strains, multiple chromosomal mutations were detected, and all except three strains had additional PMQR genes. Sequence types, ST131 and ST617 predominated among *E.* *coli* isolates, while ST607 was more common out of 12 sequence types detected among the *K.* *pneumoniae*. Fluoroquinolone resistance genes were mostly associated with the IncF plasmids.

**Conclusion:**

The ESBL-PE isolates showed high rates of phenotypic resistance towards fluoroquinolones likely mediated by both chromosomal mutations and PMQR genes. Chromosomal mutations with or without the presence of PMQR were associated with high MIC values in these bacteria strains. We also found a diversity of PMQR genes, sequence types, virulence genes, and plasmid located antimicrobial resistance (AMR) genes towards other antimicrobial agents.

## Introduction

Since the introduction of fluoroquinolones in the 1980s, these agents have been routinely used for treatment of several bacterial infections. In the recent years, ciprofloxacin has been the most consumed antibacterial agent world-wide [1]. In 2014, the World Health Organization (WHO) highlighted fluoroquinolone resistance in *Escherichia* *coli* and related organisms as a principal public health threat [2]. Reports from North America and Europe indicated that the rate of fluoroquinolone-resistant (FQR) Gram-negative bloodstream isolates exceeded 20% while in China a higher rate up to 75% has been reported [3–5]. A study from Togo, West Africa, reported that 69% of clinical isolates carried genes responsible for FQR [6]. Like other antimicrobial resistant pathogenic bacteria, FQR bacteria have negative clinical implications including treatment failure, increasing treatment costs and protracted therapy [7][7]. It has also been reported that previous colonization with FQR *E.* *coli* can lead to the spread of extended-spectrum β-lactamase-producing *Enterobacterales* (ESBL-PE) after the use of quinolone prophylaxis [9, 10].

Among Gram-negative bacteria, the primary target of quinolones is topoisomerase ii (DNA gyrase). In *Enterobacterales,* resistance to fluoroquinolones may be due plasmid mediated resistance genes or chromosomal mutations which can be found together in the same bacteria. Plasmid-mediated quinolone resistance (PMQR) genes have been reported since 1990’s [11] and in recent years a global increase in the prevalence of PMQR genes have been observed [11]. These PMQR genes include the *qnr* family genes; *qnrA,* *qnrB,* *qnrC,* *qnrD* etc., gene encoding aminoglycoside-modifying enzyme; *aac-(6′)-Ib-cr*, and antibiotic efflux pump encoding genes; *oqxAB* and *qepA* [12]. Because quinolones act by binding to enzyme–DNA complexes, which form between cleaved bacterial DNA and DNA gyrase (*gyrA,* *gyrB*) or topoisomerase IV, mutations in the chromosomal genes coding for type II topoisomerases also confer resistance to quinolones [13]. Mutations associated with quinolone resistance are located in a specific region called “quinolone resistance-determining region” (QRDR). Studies have reported multiple mechanisms of quinolone resistance [14–16].

In Dar es Salaam, Tanzania, a steady increase of FQR has been observed over time; in 2001–2002 it was reported to be 5.3% among ESBL-PE isolates obtained from children with septicemia and few years later i.e., in 2010 it was six times more (34.4%) among urinary pathogens obtained from children and adults. In 2011 the incidence of FQR was observed to be 40.5% among children in the same study settings [17–19]. Furthermore, multiple mechanisms of quinolone resistance have been reported from other continents, but there is a paucity of data from sub-Saharan Africa, particularly in Tanzania, on the molecular mechanisms responsible for quinolone resistance. Using whole genome sequencing, this study was conducted to determine quinolone resistance mechanisms among ESBL positive isolates from fecal samples from hospitalized children in Dar es Salaam, Tanzania.

## Materials and methods

### Study site and population

This was part of a prospective cross-sectional study, which was conducted from March 2017 to July 2018 in Dar es Salaam, Tanzania [20]. The study enrolled children below five years of age who were hospitalized because of fever (> 37.5 °C) at three regional hospitals: (a) Amana, (b) Temeke, (c) Mwananyamala and one tertiary hospital, (d) Muhimbili National Hospital (MNH). The study settings have been previously described in detail [20]. For this study, we randomly selected archived rectal swabs from 200 children for analysis.

### Data and specimen collection

As previously described [20], the study used Research Electronic Data Capture (REDCap), to gather demographic and clinical information including date of birth, sex, duration of fever, history of antibiotic use one month prior to admission, and history of hospitalization in the last six months. From each child, Carry Blair transport media was used to collect rectal swab which was stored at − 80 °C until the time of analysis.

### Phenotypic detection of fluoroquinolone resistant-ESBL producing *Enterobacterales* and antimicrobial susceptibility testing

First, the frozen rectal swabs were suspended in brain heart infusion (BHI) media for overnight incubation at 37 °C. Two microliter (2 µl) of BHI were then inoculated onto CHROMID® ESBL agar (BioMérieux, Marcy l’Etoile, France) and incubated for 24 h to screen for ESBL production. ESBL positive isolates were then identified by Matrix-assisted laser desorption/ionization-time of flight (MALDI-TOF) mass spectrometry (MS) using the Microflex 99 LT instrument and MALDI Biotyper 3.1 software (Bruker Daltonics, Bremen, Germany). The identified bacterial isolates were subjected to antimicrobial susceptibility testing (AST) by disk diffusion method to test for fluoroquinolone resistance using ciprofloxacin disks (5 µg). Minimum inhibitory concentration (MIC) values for all ciprofloxacin resistant isolates were then determined by E-test, using ciprofloxacin, levofloxacin and moxifloxacin strips. Interpretation of results was done based on the Clinical and Laboratory Standards Institute (CLSI) guidelines [21]. Intermediate susceptible isolates were regarded as resistant. *Klebsiella* *pneumoniae* ATCC 700603 and *Escherichia* *coli* ATCC 25922 were used as control organisms.

### Whole genome sequencing and in silico analyses

Among the FQR isolates from the phenotypic testing, 42 isolates with ciprofloxacin levels ≥ 0.5 µg/ml were randomly selected for whole genome sequencing (WGS). WGS was performed by MicrobesNG (MicrobesNG, Birmingham, UK.) using Illumina HiSeq technology [22]. DNA for sequencing was extracted using the MagNA Pure 96 DNA and Viral NA Large Volume kit (Roche Diagnostics GmbH, Mannheim, Germany) according to manufacturer’s instructions and genomic libraries were prepared using the Nextera XT DNA library preparation kit (Illumina, San Diego, CA, USA). A 150-bp paired-end sequencing was performed using the HiSeq × 10 system (Illumina). Long and short read sequences were assembled using Unicycler (v.0.4.8.0), and genome annotation was done with Prokka (v.1.14.6). Sequences were analyzed for multisequence typing (MLST), and plasmid replicon types using MLST 1.8, ResFinder [23] and Plasmidfinder software [24].

### Phylogenetic analysis

Phylogeny reconstruction was done using CSI Phylogeny (http://cge.cbs.dtu.dk/services/CSIPhylogeny/). Genomes of the isolates obtained from the present study have been submitted to Biosample Database, National Centre for Biotechnology Information (NCBI) with project accession numbers PRJNA911701 and PRJNA911976 for *E.* *coli* and *K.* *pneumoniae* respectively.

## Results

### Characteristics of study population

We performed antimicrobial susceptibility test for 142 phenotypically isolated ESBL-PE from children below five years of age. The characteristics of the children are described elsewhere [20]. Among the isolates, 64 were *E.* *coli*, 46 were *K.* *pneumoniae*, 47 were *Enterobacter* spp. and 5 were *Citrobacter* spp.

### Fluoroquinolone resistance in different species

A total of 142 ESBL-PE isolates were tested for fluoroquinolone resistance. Phenotypic resistance to fluoroquinolones, as determined by disk diffusion and E-test, was found in 97/142 (68%) of the bacterial isolates. Table 1 shows the MIC values of the three tested fluoroquinolones among different bacteria isolates. More than half of the *E.* *coli* isolates had high MIC levels (> 32 µg/ml) for the three tested quinolones: 69% (29/42) for ciprofloxacin and 68% (28/42) for both levofloxacin and moxifloxacin. Conversely, the majority of *K.* *pneumoniae* isolates had low MIC levels ranging from 0.5 µg/ml to 1 µg/ml and only 3 *K. pneumoniae* isolates had MIC levels of > 32 µg/ml (9%; 3/35). All *Enterobacter* *spp*. And *Citrobacter* *spp*. had low MIC values ranging from 05 to 3 µg/ml.

**Table 1:** Fluroquinolone MIC values among different bacterial species

MIC values (µg/ml)	Isolates					
	*E.* *coli*	*K.* *pneumoniae*	*E.* *cloacae*	*E.* *xiangfangensis*	*C.* *sedlakii*	*C.* *freundii*
	(N = 42)	(N = 35)	(N = 12)	(N = 3)	(N = 3)	(N = 2)
	n (%)	n (%)	n (%)	n (%)	n (%)	n (%)
Ciprofloxacin						
> 0.5	6 (14.3)	10 (28.6)	0 (0)	0 (0)	0 (0)	0 (0)
0.5–1	6 (14.3)	18 (51.4)	11 (91.7)	3 (100)	2 (66.7)	2 (100)
1.5–3	1 (2.4)	4 (11.4)	1 (8.3)	0 (0)	1 (1.3)	0 (0)
> 32	29 (69.0)	3 (8.6)	0 (0)	0 (0)	0 (0)	0 (0)
Levofloxacin						
> 0.5	8 (19.0)	4 (11.4)	3 (25.0)	0 (0)	0 (0)	1 (50.0)
0.5–1	5 (11.9)	26 (74.3)	9 (75.0)	3 (100)	3 (100)	1 (50.0)
1.5–3	1 (2.4)	2 (5.7)	0 (0)	0 (0)	0 (0)	0 (0)
> 32	28 (66.7)	3 (8.6)	0 (0)	0 (0)	0 (0)	0 (0)
Moxifloxacin						
> 0.5	6 (14.3)	1 (2.9)	0 (0)	0 (0)	0 (0)	0 (0)
0.5–1	6 (14.3)	29 (82.9)	9 (75.0)	3 (100)	2 (66.7)	2 (100)
1.5–3	2 (4.8)	2 (5.7)	3 (25.0)	0 (0)	1 (1.3)	0 (0)
> 32	28 (66.7)	3 (8.6)	0 (0)	0 (0)	0 (0)	0 (0)

### Plasmid mediated fluoroquinolone resistance genes and chromosomal mutations

Forty-two FQR ESBL-PE were analyzed by WGS, 20 were *E.* *coli*, 16 *K.* *pneumoniae,* 4 were *E.* *Cloacae* and 2 were *Citrobacter* *sedlakii*. Out of 42 FQR-ESBL-PE analyzed, 38 (90.5%) had PMQR genes and four (9.5%) bacteria isolates did not have any identifiable PMQR genes. As shown in Table 2. Twenty bacteria isolates (52.4%) had two or more resistance genes with the most common combination of genes being *aac(6’)-lb-cr*, *qnrB1* *and* *oqx*. The aminoglycoside acetyltransferase-coding gene *aac(6’)-lb-cr* was detected in most of the isolates (76.2%;32/42), followed by *qnrB1* (40%;17/42) and *oqx* (35.7%; 15/42). Other *qnr* genes *qnrB6* and *qnS1* were detected in 3 and 4 isolates each. Distribution of PMQR genes varied among different species. Among 20 *E.* *coli* isolates 15 had *aac(6’)-lb-cr,* (of these one had both *aac(6’)-lb-cr* and *qnrB1)*, one had *qnS1* only and four had no detectable PMQR genes. In the 16 *K.* *pneumoniae* isolates, the frequency of PMQR genes detected were; 15-*oqx,* 11-*aac(6’)-lb-cr*, 11-*qnrB1*, 3-*qnrB6* *and* 2-*qnS1.* Of note, plasmid-mediated efflux pump genes (*oqx)* and *qnrB6* genes were detected only in *K.* *pneumoniae* isolates. All the four *E.* *cloacae* isolates had both *aac(6’)-lb-cr* and *qnrB1* genes detected while one *C.* *sedlakii* had both *aac(6’)-lb-cr* and *qnrB1* genes and the second had only *aac(6’)-lb-cr* gene detected.

**Table 2:** Phenotypic and genotypic characterization of fluoroquinolone resistant isolates

Isolate number	Isolate identity	MIC values (µg/ml)			ST	PMQR genes					QRDR mutations		
		CIP	LEV	MX		*aac(6’)-lb-cr*^*a*^	*qnrB1*	*qnrB6*	*qnrS1*	*Oqx*	gyrA	ParC	ParE
407	*E.* *coli*	> 32 (R)	> 32 (R)	> 32 (R)	10	+	−	−	−	−	S83L,	S80I	S458A
											D87N		
415	*E.* *coli*	> 32 (R)	> 32 (R)	> 32 (R)	617	+	−	−	−	−	S83L,	S80I	S458A
											D87N		
451	*E.* *coli*	> 32 (R)	> 32 (R)	> 32 (R)	131	+	−	−	−	−	S83L,	S80I	I529L
											D87N	E84V	
822	*E.* *coli*	> 32 (R)	> 32 (R)	> 32 (R)	131	+	−	−	−	−	S83L,	S80I	I529L
											D87N		
868	*E.* *coli*	> 32 (R)	> 32 (R)	> 32 (R)	4981	−	−	−	−	−	S83L,	S80I	S458A
											D87N		
1185	*E.* *coli*	> 32 (R)	> 32 (R)	> 32 (R)	617	+	−	−	−	−	S83L,	S80I	I529L
											D87N		
1253	*E.* *coli*	> 32 (R)	> 32 (R)	> 32 (R)	131	−	−	−	−	−	S83L,	S80I,	I529L
											D87N	E84V	
1448	*E.* *coli*	> 32 (R)	> 32 (R)	> 32 (R)	167	+	−	−	−	−	S83L,	S80I	I529L
											D87N		
1476	*E.* *coli*	> 32 (R)	> 32 (R)	> 32 (R)	617	−	−	−	−	−	S83L,	S80I	S458A
											D87N		
1520	*E.* *coli*	> 32 (R)	> 32 (R)	> 32 (R)	131	+	−	−	−	−	S83L,	S80I	I529L
											D87N	E84V	
1637	*E.* *coli*	> 32 (R)	> 32(R)	> 32(R)	167	+	−	−	−	−	S83L,	S80I	S458A
											D87N		
2124	*E.* *coli*	> 32 (R)	> 32 (R)	> 32 (R)	617	+	−	−	−	−	S83L,	S80I	S458A
											D87N		
2130	*E.* *coli*	> 32 (R)	> 32 (R)	> 32 (R)	1193	+	−	−	−	−	S83L,	S80I	S416F
											D87N		
2163	*E.* *coli*	> 32 (R)	> 32 (R)	> 32 (R)	617	+	−	−	−	−	S83L,	S80I	S458A
											D87N		
2171	*E.* *coli*	> 32 (R)	> 32 (R)	> 32 (R)	410	+	−	−	−	−	S83L,	S80I	S458A
											D87N		
2240	*E.* *coli*	> 32 (R)	> 32 (R)	> 32 (R)	1193	+	−	−	−	−	S83L,	S80I	S416F
											D87N		
2258	*E.* *coli*	> 32 (R)	> 32 (R)	> 32 (R)	1193	+	−	−	−	−	S83L,	S80I	S416F
											D87N		
1779	*E.* *coli*	1 (R)	0.50 (R)	0.75 (R)	131	−	−	−	−	−	None	None	I529L
132	*E.* *coli*	0.75 (R)	0.38 (R)	1 (R)	34	+	+	−	+	−	None	None	None
453	*E.* *coli*	0.50 (R)	1.5 (R)	1.5 (R)	155	−	−	−	+	−	S83A	None	None
563	*K.* *pneumoniae*	> 32 (R)	> 32 (R)	> 32 (R)	38	+	+	−	−	−	None	None	None
1424	*K.* *pneumoniae*	> 32 (R)	> 32 (R)	> 32 (R)	16	−	−	−	−	+	None	None	None
2315	*K.* *pneumoniae*	3 (R)	2 (R)	3 (R)	231	−	−	−	−	+	None	None	None
2111	*K.* *pneumoniae*	2 (R)	0.50 (R)	0.75 (R)	985	+	+	−	−	+	None	None	None
2166	*K.* *pneumoniae*	1.5 (R)	0.50 (R)	0.75 (R)	348	+	+	−	−	+	None	None	None
58	*K.* *pneumoniae*	1(R)	0.75 (R)	1 (R)	607	+	+	+	+	+	None	None	None
906	*K.* *pneumoniae*	1 (R)	0.50 (R)	0.75 (R)	336	+	+	−	−	+	None	None	None
1722	*K.* *pneumoniae*	1(R)	0.38 (R)	0.75 (R)	3559	+	+	−	−	+	None	None	None
2129	*K.* *pneumoniae*	1(R)	0.50 (R)	1(R)	607	+		+	−	+	None	None	None
2149	*K.* *pneumoniae*	1 (R)	0.50 (R)	0.75 (R)	348	+	+	−	−	+	None	None	None
2155	*K.* *pneumoniae*	1 (R)	0.50 (R)	0.75 (R)	429	+	+	−	−	+	None	None	None
2241	*K.* *pneumoniae*	1 (R)	0.50 (R)	1 (R)	39	+	+	−	−	+	None	None	None
1583	*K.* *pneumoniae*	0.75 (R)	0.75 (R)	1.5 (R)	607	+	−	+	−	+	None	None	None
1912	*K.* *pneumoniae*	0.5 (R)	0.75 (R)	1 (R)	14	−	+	−	−	+	None	None	None
1925	*K.* *pneumoniae*	0.5 (R)	0.75 (R)	1 (R)	14	−	+	−	−	+	None	None	None
2142	*K.* *pneumoniae*	0.5 (R)	0.50 (R)	0.50 (R)	479	−	−	−	+	+	None	None	None
544^a^	*E.* *cloacae*	1 (R)	0.50 (R)	1 (R)		+	+	−	−	−			
645^a^	*E.* *cloacae*	1 (R)	1 (R)	1.5 (R)		+	+	−	−	−			
1501^a^	*E.* *cloacae*	1 (R)	0.75 (R)	1 (R)		+	+	−	−	−			
2167^a^	*E.* *cloacae*	0.50 (R)	0.50 (R)	1 (R)		+	+	−	−	−			
277^a^	*C.* *sedlakii*	1.5 (R)	1 (R)	2 (R)		+	+	−	−	−			
2006^a^	*C.* *sedlakii*	1 (R)	0.75 (R)	1 (R)		+	−	−	−	−			

*MIC* Minimum inhibitory concentration, *CIP* Ciprofloxacin, *LEV* Levofloxacin, *MX* Moxifloxacin, *ST* Sequence type, *PMQR* Plasmid mediated quinolone resistance, *QRDR* Quinolone resistance-determining region

^a^No point mutation database currently exists for the species

Regarding chromosomal mechanisms of quinolone resistance, QRDR mutations were observed in 19/42; 45.2% bacteria isolates, all of which were on *E.* *coli* while one *E.* *coli* strain did have any detectable QRDR mutations. Of the 19 *E.* *coli* strains with QRDR mutations, 17 had triple mutations *i.e.,* *gyrA*, *parC* and *parE*. One *E.* *coli* strain had single *gyrA* mutation and one had single *parE* mutation.

When analyzing the correlation between MIC values and the presence of plasmid/ chromosomal resistance mechanisms we observed that out of the 20 *E.* *coli* strains 17 had MIC values of > 32 µg/ml. These isolates had the triple QRDR mutations detected i.e., *gyrA*, *parC* and *parE* and most of them had dual *gyrA* mutations (S83L and D87N) as shown in Table 2. In addition, all except three of these 17 *E.* *coli* strains had PMQR genes. Three of the 20 *E.* *coli* strains had low MIC values, one had single *gyrA* mutation with no additional detectable PMQR genes, the second had a single *parE* mutation also with no additional PMQR genes and the third had 2 PMQR genes without any detectable chromosomal mutations.

In all the 16 *K.* *pneumoniae* strains we did not detect any chromosomal mutations and three strains had high MIC values of > 32 µg/ml. In these three strains, two had only one plasmid-mediated efflux pump gene (*oqx)* while the third strain had combination of PMQR genes (*aac(6’)-lb-cr* and *qnrB1*).

### Multi-locus sequence types

Through analyses of MLST profiles of *E.* *coli*, we identified eight different sequence types (STs); ST131 (n = 5), ST617 (n = 5), ST1193 (n = 3), ST167 (n = 2) and five singletons; ST10, ST34, ST155, ST410 and ST4981 (Table 3). As shown in Table 4, a total of 12 different STs were found among the *K.* *pneumoniae* isolates; ST14, ST16, ST38, ST39, ST231, ST336, ST348, ST429, ST479, ST607, ST985 and ST3559. ST607 was more prevalent than others (3/12; 25%).

**Table 3:** Sequence types, virulence genes and plasmids of 20 fluoroquinolone resistant *E.* *coli* isolates

Isolate ID	ST	Virulence genes	Plasmids	β-lactam resistance genes
1185	617	*fyu,* *gadA,* *iucC,* *iutA,* *terC,traT,* *irp2,* *iss,* *capU*	IncFII, IncFIA,IncFIB(AP001918)	***bla***_**CTX-M-15**,_ *bla*_OXA-1_
2163	617	*fyu,* *gadA,* *iucC,* *iutA,* *terC,traT,* *irp2,* *iss*	IncFII,InFIA, IncFIB(AP001918)	***bla***_**CTX-M-15**_
415	617	*fyu,* *gadA,* *iucC,* *iutA,* *terC,traT,* *irp2,* *iss*	IncFII, IncFIA, IncFIB(AP001918)	***bla***_**CTX-M-15**,_ *bla*_OXA-1_
2124	617	*fyu,* *gad,* *terC,* *traT,* *irp2,* *iss,* *capU*	IncY, IncFIA, IncFII	***bla***_**CTX-M-15,**_ ***bla***_**TEM-1B**_*,* *bla*_OXA-1_
1476	617	*fyu,* *iucC,* *iutA,* *terC,traT,* *iss,* *capU*	IncFII, IncFIA, IncFIB(AP001918), Col8282	***bla***_**CTX-M-15**_
1779	131	*fyu,* *gad,* *iucC,* *iutA,* *terC,* *traT,* *irp2,* *iss,* *chuA,* *kpSE,usp,* *kpSMII_K5,* *ompT,pap_F13,papC,sitA,ORF3,ORF4,aap,aar,aatA,afaD,agg3C,agg3D,agg5A,aggR,caf1*	IncQ1, IncFII(pRSB107)	***bla***_**TEM-1B**_
1520	131	*fyu,* *gad,* *iha,* *iucC,* *iutA,* *terC,* *traT,* *iss,* *afaA,* *afaC,* *afaD,* *astA,* *chuA,* *hrA,* *kpSE,* *kpSMIIK5,* *nfaE,* *ompT,* *pap_F43,* *papC,sat,* *senB,* *sitA,* *usp,* *yfcV*	IncFII(prSB107),IncFIB(AP001918),	***bla***_**CTX-M-15**_***,*** ***bla***_**TEM-1B**,_*bla*_OXA-1_
451	131	*fyu,* *gad,* *iha,* *iucC,* *iutA,* *terC,* *traT,* *irp2,* *iss,* *afaA,* *afaC,* *afaD,* *astA,* *chuA,* *hrA,* *kpSE,* *kpSMII_K1,* *kpSMII_K5,* *nfaE,* *ompT,* *pap_F19,* *pap_F43,* *papC,* *sat,* *senB,* *sitA,* *usp,* *yfcV*	IncFII(pRSB107), IncFIA,	***bla***_**CTX-M-15,**_ ***bla***_**TEM-1B**_*,* *bla*_OXA-1_
			IncFIB(AP001918),	
822	131	*fyu,* *gad,* *iha,* *iucC,* *iutA,* *terC,* *traT,* *irp2,* *iss,* *afaA,* *afaC,* *afaD,* *astA,* *chuA,* *hrA,* *kpSE,* *kpSMII_K1,kpSMII_K5,* *nfaE,* *ompT,pap_F19,* *pap_F43,* *papC,* *sat,* *senB,* *sitA,* *usp,* *yfcV*	IncFII(pRSB107),IncFIA,	***bla***_**CTX-M-15,**_ ***bla***_**TEM-1B**_*,* *bla*_OXA-1_
			IncFIB(AP001918),Col156	
1253	131	*fyu,* *iha,* *iucC,* *iutA,* *terC,* *traT,* *irp2,* *iss,* *chuA,* *kpSE,kpSMII_K5,* *ompT,* *pap_F43,* *sat,* *senB,* *sitA,* *usp,* *yfcV*	IncFII(pRSB107),IncFIA,	***bla***_**CTX-M-27**_
			IncFIB(AP001918),Col156	
2130	1193	*fyuA,* *gad,* *iha,* *iucC,* *iutA,* *terC,* *irp2,* *chuA,* *kpsE,* *kpsMII_K1,* *ompT,* *sat,* *senB,* *sitA,* *yfcV,* *vat,* *neuC*	IncQ1,IncFIA,IncFIB(AP001918),	***bla***_**CTX-M-15**_
2240	1193	*fyuA,* *gad,* *iha,* *iucC,* *iutA,* *terC,* *irp2,* *chuA,* *kpsE,* *kpsMII_K1,* *ompT,* *papA_F43,* *sat,* *sen* *B,* *sitA,* *yfcV,* *vat,* *neuC*	IncQ1,IncFIA,IncFIB(AP001918),Col156, Col(BS512)	***bla***_**CTX-M-15**_*,* *bla*_OXA-1_
2258	1193	*fyuA,* *gad,* *iha,* *iucC,* *iutA,* *terC,* *irp2,* *chuA,kpsE,* *kpsMII_K1,* *ompT,* *papA_F43,* *sat,* *sen* *B,* *sitA,* *yfcV,* *vat,* *neuC,* *cib*	IncQ1, IncFIB(AP001918),Incl1,Col(BS512), Col156	***bla***_**CTX-M-15**_*,* *bla*_OXA-1_
1448	167	*gad,* *terC,* *traT,* *iss,* *hrA,* *capU*	IncFII,IncFIA,Col440I, Col(BS512)	***bla***_**CTX-M-15**_***,*** ***bla***_**TEM-1B**,_ *bla*_OXA-1_*,*
1637	167	*gad,* *terC,* *traT,* *iss,* *hrA*	IncFII, IncFIA, Col440I, Col(BS512)	***bla***_**CTXM-15**_***,*** ***bla***_**TEM-1B**,_ *bla*_OXA-1_*,* *bla*_NDM-5_
868	4981	*fyuA,* *gad,* *terC,* *traT,* *irp2,* *iss*	IncFII, IncFIA,IncFIB(AP001918)	***bla***_**CTX-M-15**_
2171	410	*gad,* *terC,* *IpfA*	IncQ1,IncFIB(AP001918),IncFII(pAMA1167-NDM-5),Col(BS512)	***bla***_**CTX-M-15**_***,*** ***bla***_**TEM-1B,**_ *bla*_OXA-1_*,* ***bla***_CMY-2_
453	155	*gad,* *iroN,* *iucC,* *iutA,* *terC,* *traT,* *iss,* *cvaC,* *etsC,* *hlyF,* *IpfA,* *mchF,* *tsh*	IncFIC(FII),IncFIA,IncFIB(AP001918)	***bla***_**TEM-1B**_
132	34	*espA,* *espl,* *fyuA,* *gad,* *iha,* *iroN,* *iucC,* *iutA,* *terC,* *tir,* *traT*	IncFII(pCoo), IncY, IncHI2, IncHI2A	***bla***_**CTX-M-15**_**,** ***bla***_**TEM-1B**_*,* *bla*_ACT-7_
407	10	*fyuA,* *gad,* *iucC,* *iutA,terC,* *traT,* *irp2*	IncFIA, IncFII(pAMA1167-NDM-5)	***bla***_**CTX-M-14,**_ ***bla***_**CTX-M-24,**_ *bla*_OXA-1_

**Table 4:** *K. pneumoniae* isolates (n = 16) characterization including sequence type, wzi, plasmid and virulence profiles by WGS

Isolate ID	ST	*wzi* allele	Virulence genes	Plasmids	β-lactam resistance genes
2129	607	133	*iutA,* *tratT,* *terC,* *irp2*	IncFII(K), IncFIB(K), IncR, IncFIB(Mar)	***bla***_**CTX-M-15**_***,*** ***bla***_**SHV-65,**_ ***bla***_**TEM-1B**_
1583	607	133	*iutA,* *tratT*	IncFII(K), IncFIB(K), IncFIA(HI1), IncR	***bla***_**CTX-M-15**_***,*** ***bla***_**SHV-65,**_ ***bla***_**TEM-1B**_
58	607	133	*iutA,* *tratT*	IncFII(K), InFIB(K), IncFIA(HI1), IncR	***bla***_**CTX-M-15**_***,*** ***bla***_**TEM-1B**_***,*** ***bla***_**SHV-65**_
2166	348	83	*iutA,* *tratT,* *irp2*	IncFII(K), IncFIB(K)	***bla***_**CTX-M-15**_***,*** ***bla***_**SHV-81**_***,*** ***bla***_**SHV-110**_***,bla***_**TEM-1B**,_ *bla*_OXA-1_
2149	348	83	*iutA,* *tratT,* *irp2*	IncFIB(K)	***bla***_**CTX-M-15**_***,*** ***bla***_**SHV-81**_***,*** ***bla***_**SHV-110**_***,bla***_**TEM-1B**,_ *bla*_OXA-1_
1912	14	2	*iutA,* *tratT*	IncFII(K), IncFIB(pKPHS1)	***bla***_**CTX-M-15**_***,*** ***bla***_**SHV-28**_***,*** ***bla***_**SHV-106,**_ ***bla***_**TEM-1B**_
1925	14	2	*iutA,* *tratT*	IncFII(K), IncFIB(pKPHS1)	***bla***_**CTX-M-15**_***,*** ***bla***_**SHV-28**_***,*** ***bla***_**SHV-106,**_ ***bla***_**TEM-1B**_
1722	3559	187	*iutA,* *tratT*	IncFIB(K)	***bla***_**CTX-M-15**_***,*** ***bla***_**TEM-1B**_***,*** ***bla***_**SHV-36**_***,*** ***bla***_**SHV-80**_***,*** ***bla***_**SHV-178**_***,*** ***bla***_**SHV-193**_*,* *bla*_OXA-1_
2111	985	39	*iutA,* *tratT,* *irp2*	IncFII(K)	***bla***_**CTX-M-15**_***,*** ***bla***_**OXA-1**_***,*** ***bla***_**SHV-187,**_ ***bla***_**TEM-1B**_
2142	479	23	*iutA,* *terC*	IncFIB(K), IncY, IncHI1B, Col440I	***bla***_**CTX-M-15**_***,,*** ***bla***_**SHV-62,**_ ***bla***_**TEM-1B**_
2155	429	187	*iutA,* *tratT*	IncFII(K)	***bla***_**CTX-M-15**_***,*** ***bla***_**SHV-36**_***,*** ***bla***_**SHV-80**_***,bla***_**SHV-178**_***,bla***_**SHV-193**_***,*** ***bla***_**TEM-1B**_***,*** *bla*_OXA-1_
906	336	150	*iutA,* *tratT,* *irp2*	IncFII(K), IncFIB(K)	***bla***_**TEM-1B**_***,*** ***bla***_**SHV-94**_***,*** ***bla***_**SHV-96**_***,*** ***bla***_**SHV-172**_**,** bla_OXA-1_
2315	231	104	*iutA,* *tratT,* *irp2*	IncFII(K), IncFIB(K), IncR, Col440I, Col(MG828)	***bla***_**CTX-M-15**_***,*** ***bla***_**SHV-28**_***,*** ***bla***_**SHV-106,**_ ***bla***_**TEM-1B**_
2241	39	2	*iutA,* *fyuA,* *terC*	IncFII(K), IncFIB(K), IncFIB(Mar), IncHI1B, Col440I	***bla***_**CTX-M-15**_***,*** ***bla***_**SHV-40**_***,*** ***bla***_**SHV-56**_***,*** ***bla***_**SHV-79**_***,*** ***bla***_**SHV-85**_***,*** ***bla***_**SHV-89**_***,bla***_**TEM-1B,**_ *bla*_OXA-1_
563	38	50	*iutA,* *tratT,* *fyuA*	IncFII(K), Col440I, Col440II, Col(MG828)	***bla***_**TEM-1B**_***,*** ***bla***_**SHV-40**_***,*** ***bla***_**SHV-56**_***,*** ***bla***_**SHV-79,**_ ***bla***_**SHV-85,**_ ***bla***_**SHV-89**,_ *bla*_OXA-1_
1424	16	50	*iutA,* *tratT,* *fyuA*	IncFIB(K), IncFIA(HI1), Col440I,Col440II)	***bla***_**CTX-M-15**_***,bla***_**SHV-26**_***,bla***_**SHV-78**_***,*** ***bla***_**SHV-98**_***,*** ***bla***_**SHV-145**_***,*** ***bla***_**SHV-179**_***,*** ***bla***_**SHV-194**_***,*** ***bla***_**SHV-199,**_ ***bla***_**TEM-1B**_

Bolded β-lactam resistance genes; extended spectrum β-lactam resistance genes

All the three *K.* *pneumoniae* strains with *qnrB6* PMQR gene belonged to ST607 and had low MIC values ranging from 0.5 to 1 µg/ml. There was no other pattern or correlation established between ST types and the presence of PMQR genes, chromosomal mutations or MIC value of the other bacteria.

### Genes conferring resistance towards other antimicrobial agents

Phenotypic resistance towards gentamicin was observed in 71% (30/42) of the isolates. Of these, 90% (27/30) carried the *aac(6’)-lb-cr* gene which confers resistance to both aminoglycosides and fluoroquinolones. As shown in Table 5, we detected other antimicrobial resistance genes in 57% (24/42) of the whole genome sequenced isolates. All 24 isolates carried genes conferring resistance to aminoglycosides (100%, 24/24) and folate pathway antagonists (100%, 24/24). In most cases, the aminoglycoside-related genes appeared in combinations (96%, 23/24) except for one isolate, which carried a single gene, *aadA2*. The most detected aminoglycoside resistance gene was aminoglycoside phosphotransferase *aph* *(6)-ld*. The tetracycline resistance gene, *tetA* was detected in 83% (20/24), while the *sul2* gene, which is responsible for causing folate pathway antagonist resistance, was detected in 86% (21/24) of the isolates. Furthermore, resistance genes towards macrolides (*mphA*) and macrolides, lincosamides plus streptogramin b (*erm*(B)) were only detected in *E.* *coli* isolates, while fosfomycin resistance genes (*fosA,* *fosA5*) were only observed in *K.* *pneumoniae* isolates.

**Table 5:** Distribution of antimicrobial resistance genes other than PMQR among ESBL producing Gram negative bacteria

Isolate	Isolate	AMG	FS	Macrolide	FPA	TC	Phenicol	MLS
number	identity							
451	*E.* *coli*	*aadA5,aac(3)-lld,* *aph(6)-ld,* *aph(3')-la,* *aph(3")-lb*	–	*mph(A)*	*sul1,* *sul2,* *dfrA17*	*tet(A)*	*catB3*	–
822	*E.* *coli*	*aadA5,* *aac(3)-lld,aph(6)-ld,aph(3')-la,* *aph(3")-lb*	–	*mph(A)*	*sul1,* *sul2,* *dfrA17*	*tet(A)*	*catB3*	–
1520	*E.* *coli*	*aadA5,aac(3)-lld,* *aph(6)-ld,aph(3')-lb,* *aph(3")-lb*	–	*mph(A)*	*sul1,* *sul2,* *dfrA17*	*tet(A)*	*catB3*	–
1253	*E.* *coli*	*aadA5,* *aph(6)-ld,aph(3")-lb*	–	*mph(A)*	*sul1,* *sul2,* *dfrA17*	*tet(A)*	*-*	–
1476	*E.* *coli*	*aadA2,* *aph(6)-ld,* *aph(3")-lb*	–	–	*sul1,* *sul2,* *dfrA12*	*tet(B)*		*erm(B)*
1779	*E.* *coli*	*aac(3)-lld,aph(6)-ld,* *aph(3")-lb*			*sul1,* *sul2,* *dfrA7*	*tet(A)*	*catA1*	
453	*E.* *coli*	*aph(6)-ld,aph(3")-lb*	–	–	*sul2,* *dfrA14*	*tet(A)*		–
2124	*E.* *coli*	*aph(6)-ld,aph(3")-lb*		*mph(A)*	*sul2,* *dfrA14*	*tet(A)*	*catB3*	*erm(B)*
1185	*E.* *coli*	*aadA5,* *aph(3")-lb*	–	*mph(A)*	*sul1,* *sul2,* *dfrA17*	*tet(B)*	*catB3*	–
1448	*E.* *coli*	*aadA2,* *rmtB*		*mph(A)*	*sul1,* *dfrA12*	*tet(A)*	*catB3*	–
1637	*E.* *coli*	*aadA2,* *rmtB*		*mph(A)*	*sul1,* *dfrA12*	*tet(A)*	*catB3*	
868	*E.* *coli*	*aadA2*	–	*mph(A)*	*sul1,* *dfrA12*	–	–	*erm(B)*
2129	*K.* *pneumoniae*	*aph(6)-ld,aac(3)lla,aph(3")-lb,aadA16*	*fosA*		*sul1,* *sul2,* *dfrA27*			
563	*K.* *pneumoniae*	*aph(6)-ld,aac(3)lla,* *aph(3")-lb*	*fosA*	–	*sul2,* *dfrA14*	*tet(A)*	*catB3*	–
1722	*K.* *pneumoniae*	*aph(6)-ld,aac(3)lla,aph(3")-lb*	*fosA,*		*sul2,* *dfrA14*	*tet(A)*	*catB3*	
1912	*K.* *pneumoniae*	*aph(6)-ld,aac(3)lla,aph(3")-lb*	*fosA*		*sul2,* *dfrA14*	*tet(A)*		
1925	*K.* *pneumoniae*	*aph(6)-ld,aac(3)lla,aph(3")-lb*	*fosA*		*sul2,* *dfrA14*	*tet(A)*		
2111	*K.* *pneumoniae*	*aph(6)-ld,aac(3)lla,aph(3")-lb*	*fosA*		*sul2,* *dfrA14*	*tet(A)*	*catB3*	
1583	*K.* *pneumoniae*	*aph(6)-ld,aac(3)lla,* *aadA16*	*fosA*		*sul1,* *sul2,* *dfrA27*	*tet(D)*	*catA2*	
906	*K.* *pneumoniae*	*aph(6)-ld,aph(3")-lb*	*fosA*		*sul2,* *dfrA14*	*tet(A)*	*catB3*	–
1424	*K.* *pneumoniae*	*aac(3)-lla*	*fosA5*	–	*dfrA14*	*tet(A)*	*catA2*	–
544	*E.* *cloacae*	*aph(6)-ld,aac(3)lla,aph(3")-lb,* *aadA1*		–	*sul1,* *dfrA14*	*tet(A)*	*catA1,* *catB3*	–
1501	*E.* *cloacae*	*aph(6)-ld,aac(3)lla,aph(3")-lb,aadA1*		–	*sul1,* *sul2,* *dfrA14*	*tet(A)*	*catA1,catB3*	–
645	*E.* *cloacae*	*aph(6)-ld,aph(3")-lb,* *aadA1*		–	*sul1,* *dfrA14*	*tet(A)*	*catA1,catB3*	–

*AMG*-aminoglycosides, *FS* Fosfomycin, *FPA* folate pathway antagonist, *TC* tetracycline, *MLS* macrolide, lincosamide and streptogramin b

### Virulence genes, plasmids and beta-lactam resistance genes of *E. coli* isolates

The commonest beta-lactam resistance gene across all strains was *bla*_CTX-M-15_ (11/20), Table 3. Three out of the five ST131 *E.* *coli* isolates carried a combination of *bla*_CTX-M-15_, *bla*_TEM-1B_ and *bla*_OXA-1_. The rest of the ST131 strains carried *bla*_CTX-M-27_ or *bla*_TEM-1B_. We identified several virulence genes among the *E.* *coli* isolates and each isolate had three or more virulence genes. Among the different ST types, *E.* *coli* strains with sequence type 131 had more virulence genes compared to other STs, including those encoding for; increased serum survival (*iss*), heat-resistant agglutinin (*hrA*), fimbrial protein (*yfcV*) and plasmid-encoded enterotoxin (*senB*). The uropathogenic specific protein (usp)-encoding gene was only detected in ST131 isolates. Additionally, *terC* and *traT* were the most common virulence genes which were found across almost all different ST types. All strains harbored at least one of the three plasmids of the incompatibility group F (IncF), namely the IncFII, IncFIB and IncFIA, the most common one being IncFII. Furthermore, some of the strains harbored IncH, Col and IncX plasmids.

### Virulence genes, plasmids and beta-lactam resistance genes of *K. pneumoniae* isolates

Nine different *wzi* types were identified. The most frequently detected was *wzi*-133, which was assigned to all (3/3) ST607 isolates. Three isolates carried the *wzi-*2 allele, which encodes the type K2 capsular antigen. Regarding virulence genes, all isolates had a sidephore *iutA*, which is a ferric aerobactin receptor. Additionally, all but two (14/16) isolates carried the invasion gene *tratT*, and three isolates (ST16, ST38 and ST39) carried a receptor for the yersiniabactin system (*fyuA*). Other virulence genes detected were *irp2* and *terC*. Plasmid analysis revealed diversity of incompatibility (Inc) group plasmids (n = 8) among the isolates. The most frequent was IncFII(K) detected in 12/16 isolates followed by IncFIB(K) detected among 11/12 isolates. In addition, several variants of Col plasmids (Col440I, Col440II, ColMG828) were detected in four isolates.

### Phylogenetic analysis

**Fig. 1:**
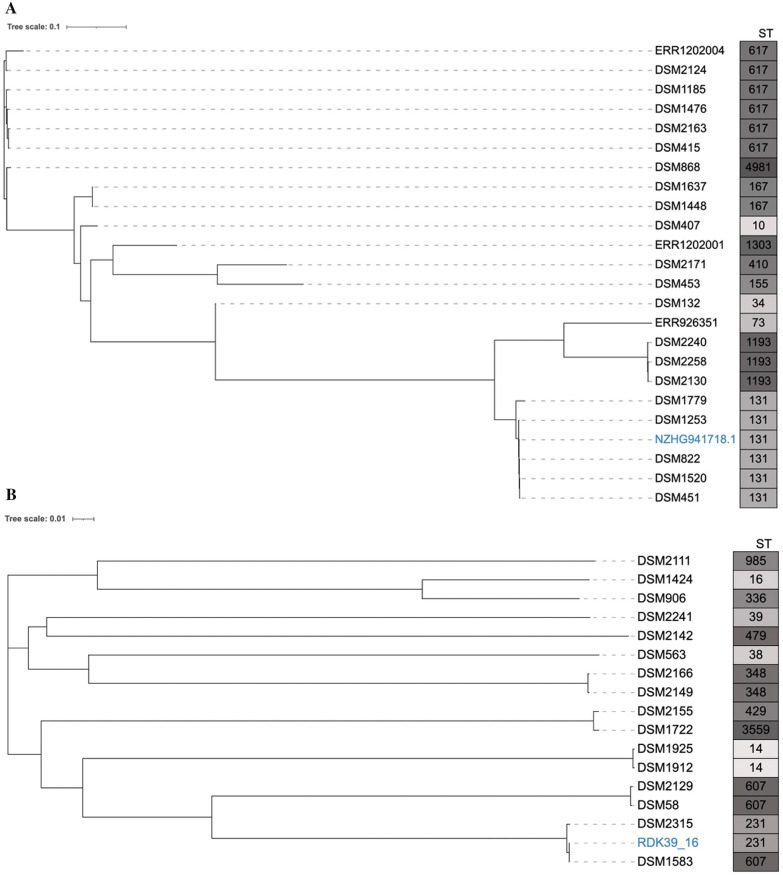
Phylogenetic tree for *E.* *coli* isolates from this study. **A** Phylogenetic analysis of 20 Fluroquinolone resistant ESBL-producing *Escherichia* *coli* rooted with *E.* *coli* NZHG941718.1 genome. Indicated in boxes are sequence types of the analyzed isolates. **B** Phylogenetic tree for *K.* *pneumoniae* isolates from this study. **B** Phylogenetic analysis of 16 Fluroquinolone resistant ESBL-producing *Klebsiella* *pneumoniae* compared with *K.* *pneumoniae* RDK39_16/NTUH-K2044 genome. Indicated in boxes are sequence types of the analyzed isolates

Whole-genome phylogenetic analysis revealed that our isolates are highly diversified, with a SNP count between genomes being 3-38590 for *E.* *coli* and 11-2128 for *K.* *pneumoniae* isolates. (Fig. 1A, B). Two *E.* *coli* isolates belonging to ST167 were closely related with SNP difference 3. Also, *E.* *coli* ST131 isolates were related with a SNP count 9–12, however these isolates were not related to the refence strain *E.* *coli* isolate ST131 isolated from United Kingdom (SNP difference 47–55). For *K.* *pneumoniae* isolates, the isolates with sequence type ST348 were somewhat closely related with a SNP difference of 11 while the SNP difference between the two ST14 isolates was 22 showing some degree of relatedness. Although having the same sequence type, *K.* *pneumoniae* ST607 isolates had no genetic relationship (SNP difference 72–15,650).

## Discussion

The present study identified a high proportion (68%) of fluoroquinolone resistance among ESBL-producing *Enterobacterales* isolates obtained from fecal samples from children below five years of age hospitalized in Dar es Salaam healthcare facilities. This proportion is high compared to what has been reported in similar settings over the past two decades [17–19]. This high proportion of resistance is concerning, considering that the use of fluoroquinolones in children is discouraged due to potential adverse effects [25]. Presumably, the high prevalence reported here might not be directly associated with the selective pressure caused by the quinolone use in this age group, but rather due to microbial transmission from adults and / or another reservoir such as the environment [26]. A report from Tanzania points out that fluoroquinolones are the most prescribed antibiotic in the country [27, 28], and it is known that exposure to quinolone even in low concentrations increases the risk for selection of resistance [26]. Therefore, the fact that expanded-spectrum cephalosporins and aminoglycosides are widely used among Tanzanian children may also contribute to the high prevalence reported here. Furthermore, previous reports have shown genetic linkage between resistance to beta-lactams and quinolones in ESBL-producing isolates, whereby quinolone resistance rate is found to be high [29]. This is because PMQR genes are frequently found on the same resistance plasmids as genes conferring ESBL and aminoglycosides [30]. Some studies have reported high rate of quinolone resistance among ESBL-positive isolates [29]. In this study, all bacteria isolates are ESBL-positive, and this could partly explain the high rate of quinolone resistance found.

Nonetheless, we cannot rule out the difference in timeline between our study and previous studies and differences in settings rural/urban, hospital/community as potential contributors to the observed difference. Comparable proportions of fluoroquinolone-resistant *Enterobacteriaceae* have also been documented in other parts of the world [31], while lower resistance rates have been reported in Kenya [32] and in Ethiopia [31]. The difference may be attributed by numerous factors such as varying third generation cephalosporins use in the different settings. The observed difference highlights the importance of relevant local data. We observed variation in the rate of fluoroquinolone resistance among different bacteria species with high rate in *K.* *pneumoniae* compared to *E.* *coli*. Fluoroquinolone resistance is reported to be mainly due to chromosomal mutations in the genes encoding type ii topoisomerases. This process is usually sequential, the appearance of the first mutation in *gyrA* favors the appearance of new mutations in *parC*, and additional number of mutations causes an increase in the ciprofloxacin MIC above 2 mg/l [33]. Furthermore, studies have shown an interplay between plasmid and chromosomal mediated quinolone resistance where combination of PMQR and QRDR increases MIC values [34]. Of note, majority of *E.* *coli* strains in this study had high MIC values of above > 32 µg/ml. The high MIC values in *E.* *coli* strains could partly be due to high number of mutations as well as presence of both PMQR genes and chromosomal mutations in the same strains. On the other hand, most *K.* *pneumoniae* strains had low MIC values and none of the strains had any detectable chromosomal mutations, which could be the reason behind low MIC values in these strains.

In this study we report detection of different PMQR genes, the most predominant one being *aac* *(6̍)-Ib-cr* gene which is also prevalent in other parts of Tanzania [35, 36]. The predominance of the *qnrB* gene has previously been reported in bacterial strains from Africa [37]. Contrary to our findings, Mshana et al. [36] reported the predominance of another plasmid mediated resistance gene *qnS1* in Mwanza, among children and adults in the northern part of Tanzania. This difference may be attributed to different geographical locations or different study populations between the two studies. Previous studies have shown that PMQR genes are usually found in plasmids which also carry resistance genes towards cephalosporins (ESBL), aminoglycosides, chloramphenicol, rifampicin, sulphonamides, tetracycline and trimethoprim [38].

Co-localization of antibiotic resistance genes on the same mobile genetic element such as plasmids is of great concern because it makes the transfer and spread of resistance genes between and within bacteria species easy. Some studies have demonstrated PMQR genes are easily transferable by conjugation [38–40].

Noteworthy is the phenotypic expression observed in isolates with combination of *qnrB1* *and* *aac(6’)-lb-cr* genes. We observed that isolates which had both *qnrB1* *and* *aac(6’)-lb-cr* genes had lower MIC levels. The two genes have been known to cause low level resistance which might not reach the breakpoints for phenotypic resistance; however, this finding poses a risk since these genes, especially when in combination, can facilitate selection of higher-level quinolone resistance.

The presence of genes encoding resistance to aminoglycosides, folate pathways inhibitors, fosfomycin, macrolides, lincosamide plus streptogramin b were also identified in the present study. Of concern is the detection of fosfomycin resistance genes among *K.* *pneumoniae* isolates. Faced with the growing AMR problem and shortage of new antimicrobial agents, there is renewed interest in older antibiotics such as fosfomycin that is currently used as a last-resort, rescue treatment against multidrug‐resistant bacteria especially ESBL-PE and carbapenemase‐producing *Enterobacteriaceae* [41]. Hence detection of these genes in the isolates is worrisome and warrants further studies.

The predominance of *E.* *Coli* ST617 and ST131 observed in the current study has also been reported by others [42, 43]. Similar to previous reports these strains harbor *bla*_CTX-M-15_ genes in multiple IncF, which is considered pandemic, as they have been detected in several parts of the world and in bacteria of different origins and sources [44]. A rare IncY plasmid was observed in two isolates: ST34 and ST617 carrying *bla*_CTX-M-15_*,* *bla*_OXA-1_and *bla*_TEM-1B_. This plasmid was also detected by Mshana et al. among clinical isolates in Mwanza, Tanzania [45]. Moreover, we report a high-risk clone ST155 which has not been reported in Tanzania. Furthermore, we report correlation of ST607 *K.* *pneumoniae* isolates which was the most prevalent sequence type with PMQR gene *qnrB6.* The predominance of plasmid mediated *qnr* genes has also been reported in Egypt [14]. Our isolates were highly diversified, with only few showing some degree of relatedness. Those that were clonally related were isolated from children attending the same hospital.

Fluoroquinolones have broad spectrum of activity and are effective in treating a wide spectrum of infections caused by aerobic Gram-negative organisms, particularly *Enterobacteriaceae*, and provide additional activity against Gram-positive organisms [1]. Although, fluoroquinolones have restricted use in pediatric population [25], the current trend of increase resistance towards other antimicrobial agents such as cephalosporins, cotrimoxazole and aminoglycosides has led to an increased use of fluoroquinolone in children as alternative therapeutic agent. It is therefore important to monitor the trend and resistance mechanisms of fluoroquinolone in settings where this antimicrobial is the remaining relatively affordable treatment alternative.

## Conclusions

This study indicates that there is a high prevalence of fluoroquinolone resistance caused by both chromosomal mutations and plasmid mediated genes (PMQR) among ESBL producing Enterobacterales. We report differences in MIC values towards fluoroquinolones among different bacteria species. High number of chromosomal mutations with or without the presence of PMQR genes was associated with increased MIC values in these bacteria strains. A variety of PMQRs were detected, but the most predominant ones were *aac(6′)-Ib-cr*, *qnrB1* and *oqx*. The variety of different fluoroquinolone resistance genes detected in this single study should be taken into account when designing molecular epidemiological surveys to determine the mechanisms responsible for observed fluoroquinolone resistant phenotypes.

## Data Availability

All data generated or analyzed during this study are included in this manuscript and its Additional file.

## Abbreviations

ATCC: American Type Culture Collection
CLSI: Clinical Laboratory Standard Institute
DNA: Deoxyribonucleic acid
ESBL-PE: Extended spectrum beta-lactamase- producing Enterobacterales
FQR: Fluroquinolone resistant
MIC: Minimum inhibitory concentration
MLST: Multi locus sequence typing
MNH: Muhimbili National Hospital
PMQR: Plasmid mediated quinolone resistance genes
QRDR: Quinolone resistance determining regions
spp: Species
ST: Sequence type
WGS: Whole genome sequencing
WHO: World Health Organization
